# Supplementary material for: Virtual Monochromatic Images from Dual-Energy Computed Tomography Do Not Improve the Detection of Synovitis in Hand Arthritis
Source: Diagnostics (Basel). 2022 Aug 4;12(8):1891. doi: 10.3390/diagnostics12081891 (PMC9406820; doi:10.3390/diagnostics12081891)
Supplement: Supplementary file 1 [file diagnostics-12-01891-s001.zip › Supplement Table S1 VMI.pdf]

Supplementary Table S1: Results of the contingency table analysis. SE = sensitivity, SP = specificity, PPV = positive predictive value, NPV = negative predictive value. In brackets 95% CI = 95% confidence interval of the difference. + synovitis / tenosynovitis positive, - synovitis / tenosynovitis negative.

Patient level								Joint/Tendon level						
	MSUS +	MSUS -							MSUS +	MSUS -				
**80 kVp +**	26	3	**SE**	0.93 (0.77 – 0.99)	**PPV**	0.90 (0.74 – 0.96)		**80 kVp +**	90	54	**SE**	0.58 (0.50 – 0.66)	**PPV**	0.63 (0.54 – 0.70)
**80 kVp -**	2	2	**SP**	0.4 (0.07 – 0.77)	**NPV**	0.5 (0.09 – 0.91)		**80 kVp -**	65	484	**SP**	0.90 (0.87 – 0.92)	**NPV**	0.88 (0.85 – 0.90)
**135 kVp +**	22	0	**SE**	0.79 (0.60 – 0.90)	**PPV**	1.0 (0.85 – 1.0)		**135 kVp +**	78	27	**SE**	0.50 (0.43 – 0.58)	**PPV**	0.74 (0.65 – 0.82)
**135 kVp -**	6	5	**SP**	1.0 (0.57 – 1.00)	**NPV**	0.45 (0.21 – 0.72)		**135 kVp -**	77	511	**SP**	0.95 (0.93 – 0.97)	**NPV**	0.87 (0.84 – 0.89)
**50 keV +**	24	2	**SE**	0.86 (0.69 – 0.94)	**PPV**	0.92 (0.76 – 0.99)		**50 keV +**	79	35	**SE**	0.51 (0.43 – 0.59)	**PPV**	0.69 (0.60 – 0.77)
**50 keV -**	4	3	**SP**	0.6 (0.23 – 0.93)	**NPV**	0.43 (0.16 – 0.75)		**50 keV -**	76	503	**SP**	0.93 (0.91 – 0.95)	**NPV**	0.87 (0.84 – 0.89)
**70 keV +**	27	3	**SE**	0.96 (0.83 – 0.99)	**PPV**	0.9 (0.74 – 0.97)		**70 keV +**	94	56	**SE**	0.61 (0.53 – 0.68)	**PPV**	0.63 (0.55 – 0.70)
**70 keV -**	1	2	**SP**	0.4 (0.07 – 0.77)	**NPV**	0.67 (0.12 – 0.98)		**70 keV -**	61	482	**SP**	0.90 (0.87 – 0.92)	**NPV**	0.89 (0.86 – 0.91)
**Joint level**								**Tendon level**						
	MSUS +	MSUS -							MSUS +	MSUS -				
**80 kVp +**	65	27	**SE**	0.56 (0.47 – 0.64)	**PPV**	0.71 (0.61 – 0.79)		**80 kVp +**	25	27	**SE**	0.66 (0.50 – 0.79)	**PPV**	0.48 (0.35 – 0.61)
**80 kVp -**	52	219	**SP**	0.89 (0.85 – 0.92)	**NPV**	0.81 (0.76 – 0.85)		**80 kVp -**	13	265	**SP**	0.91 (0.87 – 0.94)	**NPV**	0.95 (0.92 – 0.97)
**135 kVp +**	55	13	**SE**	0.47 (0.38 – 0.56)	**PPV**	0.81 (0.70 – 0.88)		**135 kVp +**	23	14	**SE**	0.61 (0.45 – 0.74)	**PPV**	0.62 (0.46 – 0.76)
**135 kVp -**	62	233	**SP**	0.95 (0.91 – 0.97)	**NPV**	0.79 (0.74 – 0.83)		**135 kVp -**	15	278	**SP**	0.95 (0.92 – 0.97)	**NPV**	0.95 (0.92 – 0.97)
**50 keV +**	59	25	**SE**	0.50 (0.42 – 0.59)	**PPV**	0.70 (0.60 – 0.79)		**50 keV +**	20	10	**SE**	0.53 (0.37 – 0.68)	**PPV**	0.67 (0.49 – 0.81)
**50 keV -**	58	221	**SP**	0.90 (0.85 – 0.93)	**NPV**	0.79 (0.74 – 0.84)		**50 keV -**	18	282	**SP**	0.97 (0.94 – 0.98)	**NPV**	0.94 (0.91 – 0.96)
**70 keV +**	72	35	**SE**	0.62 (0.52 – 0.70)	**PPV**	0.67 (0.58 – 0.75)		**70 keV +**	22	21	**SE**	0.58 (0.42 – 0.72)	**PPV**	0.51 (0.37 – 0.65)
**70 keV -**	45	211	**SP**	0.86 (0.81 – 0.90)	**NPV**	0.82 (0.77 – 0.87)		**70 keV -**	16	271	**SP**	0.93 (0.89 – 0.95)	**NPV**	0.94 (0.91 – 0.97)
